# Effectiveness of workplace cancer screening interventions: a systematic review

**DOI:** 10.1186/s12885-024-12649-0

**Published:** 2024-08-12

**Authors:** Hsu Myat Mon, Kathryn A. Robb, Evangelia Demou

**Affiliations:** 1 College of Social Sciences, University of Glasgow, G12 8QQ Glasgow, Scotland; 2 School of Health and Wellbeing, University of Glasgow, G12 8QQ Glasgow, Scotland; 3 MRC/CSO Social and Public Health Sciences Unit, School of Health and Wellbeing, University of Glasgow, G12 8QQ Glasgow, Scotland; 4 Department of Preventive and Social Medicine, Faculty of Medicine, Chulalongkorn University, 1873 Rama IV Road, Khwaeng Pathum Wan, Khet Pathum Wan, Krung Thep Maha Nakhon, Bangkok, 10330 Thailand

**Keywords:** Cancer screening, Screening promotion, Screening uptake, Colorectal cancer, Breast cancer, Cervical cancer, Workplace intervention, Systematic review, Participation

## Abstract

**Introduction:**

Cancer cases are rising globally, with a noticeable rise in younger adults. Screening and early detection are effective in decreasing mortality. Workplaces can play a role in promoting cancer screening uptake. This systematic review investigated the effectiveness of workplace breast, lung, colorectal, and cervical cancer screening interventions, and the factors impacting their effectiveness.

**Methodology:**

Six databases (Embase, Medline, Web of Science, CINAHL, Cochrane Library, Scopus) were searched, and cancer screening promotion and cancer screening uptake was analysed using effect direction plots. Magnitude of effectiveness (i.e., change in knowledge or screening rate) was also evaluated.

**Results:**

In total, 13,426 articles were identified. After screening and applying the eligibility criteria, 21 articles were included in the analysis. A positive effect direction was seen for all workplace cancer screening promotion interventions. Magnitude of effectiveness for cancer screening promotion interventions resulted in a > 30% change in knowledge or screening uptake in 4/7 of breast cancer, in 3/4 of cervical cancer and 1/3 colorectal cancer screening promotion interventions. For workplace cancer screening uptake interventions, a positive effect direction was observed for the majority (18/22). Cancer screening uptake interventions showed a > 30% change in magnitude of screening rate in 4/7 breast cancer, 5/10 colorectal cancer and in 1/5 cervical cancer workplace interventions. No studies for lung cancer were eligible. Factors positively influencing effectiveness included an interest in health and previous healthcare use, while fear of cancer and embarrassment of screening negatively influenced effectiveness.

**Conclusion:**

Workplace cancer screening promotion and uptake interventions can effectively improve cancer screening knowledge and increase uptake of screening tests.

**Supplementary Information:**

The online version contains supplementary material available at 10.1186/s12885-024-12649-0.

## Introduction

Screening for cancer and precancerous lesions can improve the chance of recovery and slow progression of the disease if detected early [1–3]. Cancer screening, however, is not without side effects, and therefore, routine cancer screening tests with the lowest harm and highest benefits are recommended for specific age groups and time intervals [2, 4] (Appendix A). Despite the recommendations and programs in place, screening coverage varies widely, and even high-income countries struggle to increase and maintain screening coverage. In the United States (U.S.), median screening rates in 2020 for mammography and colorectal cancer were 71% (eligible ages: 50 to 74 years) and 59% (eligible ages: 45 to 75 years), respectively [5]. In England, 68.7% took up breast cancer screening (eligible ages: 50 to 71 years), and 65.9% (eligible ages: 60 to 74 years) were adequately screened for colorectal cancer between 2019 and 2020 [6].

Even though the prevalence of most cancers is higher in older populations, it can occur at any age [7]. A noticeable shift in cancer incidence is seen as cancer becomes more common in younger generations [8]. In 2020, new cancer cases for all cancers in the working-age population (15–69 years) were 11 million, more than half of the total diagnoses worldwide [8]. The United States Preventive Services Task Force (USPSTF) reported that from 2000–2 to 2014–16, a rise of ~ 15% was recorded in colorectal cancer incidence in people aged 40–49 years [9].

Along with this trend, the burden of cancer on the working population becomes more extensive, impacting every aspect of their lives [10]. Cancer can heavily impact work ability [11–15], financial stability [10], and productivity [11–15], resulting in difficulty securing and maintaining employment for cancer patients or those recovering [16, 17]. Further, those returning to work may endure additional challenges, such as discrimination, bias, or even layoffs if they are considered incapable of performing their jobs effectively [18]. It is evident that the burden of cancer extends beyond the individual, to their families, employers and society as a whole [16].

Workplaces, as a setting, can affect change in public health and in individual behaviours [19], and can be used to strengthen cancer screening engagement [20, 21]. Workplaces can provide easy and regular access to people from various sociodemographic and economic positions [20] and influence screening behaviour [22] by providing positive social norms, a convenient setting for screening, removing structural barriers for employees and promoting health education [19, 23]. Workplace incentives can also motivate employees to take up screening services [21]. Employers on their side, have motives to implement interventions targeting employee wellbeing, as they have a responsibility to protect and prioritise the health of their employees by implementing health and safety regulations and by promoting healthy behaviours [24]. For working environments that are considered to be exposing their employees to known cancer risks, including carcinogens, sedentary lifestyles, psychological distress, and shift work, employers and organizations may be considered partially responsible for providing cancer screening [25].

To our knowledge, workplace cancer screening intervention studies to date have only examined single workplaces, or single or two cancers at a time. Therefore, this study aims to undertake a systematic review to assess the effectiveness of workplace cancer screening promotion and cancer screening uptake interventions, as well as the factors that can facilitate or hinder engagement, uptake and the effectiveness of these interventions.

## Methodology

The protocol for this review was registered in PROSPERO (CRD42022334827).

Research Questions:

1. Are workplace cancer screening promotion interventions and cancer screening uptake interventions effective?

2. What are the factors that influence engagement, uptake and the effectiveness of these interventions?

### Workplace cancer screening promotion and screening uptake interventions

We focus on cancer screening promotion and screening uptake interventions for four common types of cancer with recommended screening tests by the USPSTF: breast, lung, colorectum, and cervix uteri [9, 26–28]. Prostate cancer screening, although the prevalence is high, was not included as this is not recommended for population-based screening [29]. During the literature review, we considered the screening recommendations by reputable organizations including the World Health Organization, the United States Preventive Services Task Force (USPSTF), the National Health Service of the United Kingdom and the American Cancer Society. In the end, we chose to use the USPSTF recommendations as the base guideline since it has the broadest and most clear range of recommended ages and screening investigations, with regular updates. Workplace cancer screening promotion interventions were defined as interventions promoting knowledge and information on existing cancer screening services, without offering physical screening tests by workplaces or researchers. This may promote the use of existing national screening programs or individual out-of-pocket screening uptake. Examples of cancer screening promotion interventions are health talks on breast cancer screening at workplaces and allowed paid time off to attend screening. Cancer screening promotion interventions were included in the review as some workplaces may only be able to promote cancer screening because firstly, the availability of cancer screening services varies depending on each country’s health system (e.g. in the UK, National Health Service (NHS) screening services are available to all eligible citizens)[30], and secondly, associated costs may render screening tests prohibitive for workplaces.

Workplace cancer screening uptake interventions were defined as interventions in which employees were offered cancer screening services by workplaces or by researchers in workplaces as another screening opportunity additional to the national programs or individual screening in the private sector (e.g., providing cervical cancer self-screening test kits to the employees at work). The workplace interventions included could be organised by the employer or by an external agency but supported by the workplace.

### Search strategy

The search strategy was generated by combining three core concepts: (i) workplace settings and/or interventions organised by workplaces; (ii) cancer; and (iii) the four sites of common cancers and screening tests. Search terms were developed for each concept, using truncations, Boolean and proximity operators, and Medical Subject Headings (MeSH). Pilot searches were conducted to balance a good level of sensitivity and specificity in identifying relevant articles. The search was performed in six databases: Embase (Ovid), Medline (Ovid), Web of Science, Cumulative Index to Nursing and Allied Health Literature (Table 1 and Appendix B).

**Table 1:** Search strategy consulted in Embase (Ovid)

((((work* OR job* OR employ* OR occupation*) adj3 (health* OR effect* OR program* OR promot* OR intervention* OR diagnos* OR screen* OR campaign* OR polic* OR service* OR initiative* OR "cancer awareness" OR scan*)).ti,ab OR occupational health [mesh] OR occupational health services [mesh] OR occupational medicine [mesh]) AND ((cancer* OR malignan* OR neoplas* OR tumo$r*).ti,ab OR cancer diagnosis [mesh] OR malignant neoplasm [mesh] OR malignant neoplastic disease [mesh] OR neoplasm [mesh]) AND ((breast* OR lung* OR colorect* OR colon OR rect* OR bowel OR intestinal OR cervi* OR colonoscop* OR sigmoidoscop* OR "computed tomography colonograph*" OR "f$ecal occult blood test*" OR "f$ecal immunochemical test*" OR "stool DNA test*" OR "stool test*" OR "double contrast barium enema" OR mammogra* OR "breast exam*" OR "breast self-exam*" OR "low dose computed tomograph*" OR "chest X-ray*"OR "cervical cytolog*" OR "pap test*" OR "pap smear" OR "high-risk human papillomavirus*" OR hpv* OR hrhpv* ).ti,ab) AND Limited to publication years of 2010 to current (8th April 2024), English language, Human studies.)	

### Inclusion and exclusion criteria

Papers were included if they: (i) presented on cancer screening promotion and screening uptake interventions delivered in workplaces and/or organised by workplaces; (ii) presented on interventions performed on working adults; (iii) were published between 2010 and the date of search (8th April 2024); and (iv) were written in English. In addition, (vi) interventions had to focus on at least one of the four types of cancer: breast, lung, colorectal, and cervical cancers, and (vii) the screening tests promoted or offered had to be recommended by the USPSTF [4]. The search was not restricted to geographical regions or study designs to obtain an inclusive overview of interventions. Studies did not have to specifically examine occupational cancers e.g. due to exposure to known carcinogens nor did the intervention have to only be in place to adhere to regulatory compliance.

Papers were excluded if they: (i) studied interventions in settings other than workplaces or that were not organised or supported by workplaces; (ii) were not on humans and not on working adults; (iii) were not published within the timeline of 2010 to the date of search (8th April 2024); (iv) were not written in English; (v) were not peer-reviewed studies, and were conference abstracts or grey literature; (vi) measured outcomes other than the targeted ones or interventions on any other cancer types; and (vii) were promoting or offering screening tests not recommended by the USPSTF (Appendix A).

### The process of identifying articles

The selection process was carried out by two independent reviewers, with the first reviewer reviewing 100% of the articles and the second reviewer screening 10% of the articles. Disagreements were resolved by discussion and all final full texts were agreed by two reviewers. The web application “Rayyan” was used to deduplicate, screen title, abstract and full text, as well as to record the codes and reasons for exclusion [31].

### Quality and risk of bias assessment

Quality assessment and risk of bias assessment was conducted by the first reviewer and a second reviewer assessed 10% of the studies. Quality assessment was done using the Consolidated Standards of Reporting Trials: 2010 statement (CONSORT) for randomised controlled trials and the Transparent Reporting of Evaluations with Nonrandomized Design (TREND) for non-randomised controlled trials [32, 33]. Individual items were rated as 1, 0.5, and 0 for each item. Scores were added and papers were rated high quality if the score was ≥ 80% of the maximum possible score, moderate quality for scores between 60 – 79% and low quality for scores < 60% [34, 35].

The revised version of the Cochrane Risk of Bias tool (ROB 2) was employed to assess randomised controlled trial (RCTs) and the Risk Of Bias In Non-randomised Studies—of Interventions (ROBINS-I) for non-randomised studies [36, 37]. “The Critical Appraisal Skills Programme (CASP) tool for Evaluating Qualitative Research” was used for appraising risk of bias for qualitative Studies [38]. Disagreements between the reviewers were resolved by discussion.

### Data extraction and data analysis

Data extraction included: (i) study characteristics; (ii) participant demographics; (iii) characteristics of workplace intervention; (iv) outcomes (change in knowledge or screening rate and factors impacting the effectiveness of interventions); and (v) other discussions and recommendations.

Our primary outcome of interest was intervention effectiveness. This was translated as effectiveness of the interventions in increasing knowledge or increasing subsequent screening rates for cancer screening promotion interventions and increasing screening uptake rates for workplace cancer screening uptake interventions. A meta-analysis could not be carried out due to the heterogeneity in types of intervention, populations addressed, study designs and outcome measures. Therefore, a narrative analysis and evidence synthesis was done using effect direction plots to assess intervention effectiveness [39]. Effectiveness of cancer screening promotion interventions was assessed by either a statistically significant change in knowledge pre/post intervention or between intervention and control groups after interventions. For the cancer screening uptake interventions, effectiveness is assessed by statistically significant changes in the screening rates/percentage taking up screening pre/post intervention or between intervention and control groups after interventions. In the plot, an upward arrow “▲” represents a positive impact, a downward arrow “▼” shows negative impact, and sideways arrows “◄►” denote no change/mixed effects/conflicting findings. In addition to intervention effectiveness, the magnitude of effectiveness was also estimated [34]. This was calculated by (**a**) comparing intervention and control groups (result of study group minus result of control group) for two-group interventions, (**b**) comparing before and after tests (screening utilization after intervention minus screening utilization before intervention) for one-group pre-test post-test interventions, and (**c**) using the absolute number of screening uptake for one-group post-test only interventions. Specific cut-off points delineating an effective magnitude or minimum levels of change in screening rates that lead to clinically significant differences have not been reported [20, 35, 40]. Therefore, for this study, the magnitude of effectiveness was categorized using a large arrow “▲” if the change in rate of cancer screening tests or improvement in knowledge of cancer was above 30%, medium arrow “▲” if the above changes were between 5–30%, and small arrow “▲” if the changes were less than 5%. GraphPad (https://www.graphpad.com/quickcalcs/binomial1/) was used to calculate the Sign test (two-tailed p-value) for each outcome domain by calculating the number of interventions with positive and negative effect directions (*p*-value = 0.5). Factors positively or negatively influencing workplace screening promotion and cancer screening uptake interventions that were reported in the original studies, either from the regression models, intervention evaluations or qualitative responses, were extracted as secondary outcomes.

## Results

**Fig. 1:**
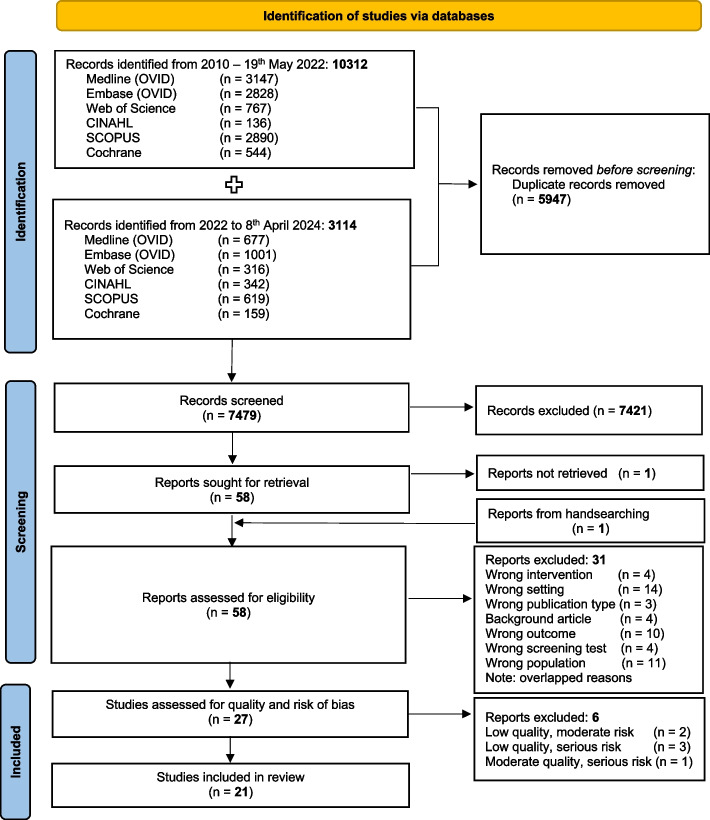
Flow diagram of the selection process of included studies

Figure 1 presents the flow diagram of study selection. A total of 13,426 articles were identified through two searches covering the period from 2010 to 8th April 2024. 7479 articles were screened for title and abstract, and 58 articles were screened for full texts. This resulted in 27 articles taken forward for quality and risk of bias assessment.

### Quality assessment and risk of bias assessment

After quality and risk of bias assessments, six articles were excluded for low quality or serious risk of bias (Appendices C, D and K). Four articles promoted and offered screening tests that are not recommended by the USPSTF [26] (i.e. breast self-examination (BSE) and clinical breast examination (CBE)) and were therefore excluded from our review. Finally, 21 articles were included for analysis.

Overall, among the twenty-one included articles, eight articles were of high quality [41–48] and thirteen of moderate quality [49–59] (Table 2 and Appendix C). Fourteen articles had low risk of bias [42, 43, 45–49, 51, 52, 55, 57, 58, 60, 61], and seven studies had moderate risk of bias [41, 44, 50, 53, 54, 56, 59] (Table 2, Appendix D.1 and D.2).

**Table 2:** Characteristics of Included Studies and Interventions

**Study ID**	**Quality of studies**	**Risk of bias of studies**	**Study design**	**Type of cancer**	**Type/Name of workplace**	**Type of intervention- Screening promotion or Screening uptake, (Type of screening test)**	**Setting of intervention, incentives involved**	**Country**
Abdullah et al. [42]	High	Low	Randomized controlled trial	Cervical	40 National Secondary Schools	Screening promotion (Invitation letter, pamphlet, reminder phone call (Pap test)	Workplace	Malaysia
Bardach et al. [55]	Moderate	Low	Controlled before-after study	Colorectal	Intervention group—Internal Revenue Agency of the Province of Buenos Aires (ARBA) Control group—Public employees of the Province of Buenos Aires and their families but from other dependencies (Non-ARBA)	Screening promotion (Brochures, advertisements, banners, social network posts, lectures, workshops, video interviews) + Screening uptake (Financial coverage) (iFOBT kits)	Workplace	Argentina
Behnke et al. * [48]	High	Low	Uncontrolled before-after study (Mixed method of non-RCT and qualitative approach)	Cervical	District Hospital in Ghana	Screening uptake (Free screening test kits) (HPV self-sampling test kits)	Workplace (Drink, snack and small gift for household use)	Ghana
Bernstein et al. * [44]	High	Moderate	Uncontrolled before-after study	Colorectal + Cervical + Breast	Rally Health (A Digital Health Company)	Screening uptake (Financial coverage through health savings account, health reimbursement account, health incentive account, gift cards) (Screening tests—not specified)	Workplace (Financial incentives)	USA
Callison et al. [61]	Moderate	Low	Uncontrolled before-after study (Quasi-experimental)	Colorectal + Breast	Large, private-sector employers	Screening promotion (Allowed paid time off work) (colonoscopy, CT colonography, flexible sigmoidoscopy, FIT, double-contrast barium enema, guaiac FOBT, and stool DNA test, and mammography)	Workplace	USA
Cuellar et al. [41]	High	Moderate	Controlled before-after study	Colorectal + Cervical + Breast	39 Workplaces (15 intervention worksites and 14 control worksites)	Screening uptake (Financial coverage through cash rewards, premium reductions, or gift cards) (Screening tests—not specified)	Workplace (Financial incentives)	USA
Eljack et al. [58]	Moderate	Low	Randomized controlled trial	Cervical	13 Secondary schools	Screening promotion (Health education session and pamphlets) (Screening tests—not specified)	Workplace	Qatar
Esmat Heydari, Azita Noroozi [57]	Moderate	Low	Randomized controlled trial	Breast	21 Elementary schools	Screening promotion (Education session, multimedia education, reminder phone call) (Mammography)	Workplace (group-based approach) and electronic (multimedia approach)	Iran
Fernandez-Esquer et al. [43]	High	Low	Uncontrolled before-after study	Breast + Cervical	59 Nail salons	Screening promotion (Education session, brochures, navigation services) (Mammography and Pap test)	Workplace (Financial incentives)	USA
Hannon et al. * [54]	Moderate	Moderate	Uncontrolled post-test only study	Colorectal	Intervention worksites—6 worksites from the education, health services, and manufacturing industries Control worksites—7 worksites (not specified on type of industries)	Screening promotion (Seminars) + Screening uptake (Free screening test kits) (FIT)	Workplace (Drawing for $20 Starbucks gift card to each worksite)	USA
Hing et al. * [51]	Moderate	Low	Uncontrolled before-after study	Breast	Changi General Hospital	Screening promotion (Health Talk) (Mammography)	Workplace	Singapore
Hui et al. [46]	High	Low	Uncontrolled before-after study	Colorectal + Breast + Cervical	State of Kansas	Screening promotion (Online health risk assessment) + Screening uptake (Financial coverage) (Screening tests—not specified)	Electronic (internet-based portal) (Financial incentives)	USA
Jensen et al. * [49]	Moderate	Low	Randomized Controlled Trial	Colorectal	6 hospitals and 2 manufacturing plants	Screening promotion (Pamphlets) + Screening uptake (Financial coverage) (Colonoscopy)	Electronic (computer-based health message)	USA
Ma et al. [50]	Moderate	Moderate	Controlled before-after study	Breast	6 product manufacturing sites and 2 research institutes	Screening promotion (Education session, group discussion, handout, navigation services, release time) + Screening uptake (Financial coverage) (Mammography)	Workplace	China
O'Keefe et al. [53]	Moderate	Moderate	Uncontrolled post-test only study	Colorectal	University of Alabama in Huntsville (UAH) and University of South Alabama (USA)	Screening promotion (Poster, postcard) + Screening uptake (Free screening test kit) (FIT)	Workplace (Drawing of $500 gift card at each uni and T-shirt, hats and pens in USA)	USA
Ozerdogan et al. [56]	Moderate	Moderate	Uncontrolled before-after study	Breast	The Eskişehir Osmangazi University	Screening promotion (Educational session, booklet, navigation) + Screening uptake (Appointment at national program) (Mammography)	Workplace	Turkey
Rafie et al. [52]	Moderate	Low	Uncontrolled before-after study	Colorectal	Virginia Cooperative Extension	Screening promotion (Seminars, communication plan, peer champion) + Screening uptake (Financial coverage) (Screening tests—not specified)	Workplace and electronic (internet-based)	USA
Shepherd et al. [47]	High	Low	Controlled post-test only study	Colorectal	The Metropolitan Nashville Public Schools system	Screening promotion (Educational letter, consultation with nurse practitioner) + Screening uptake (Free test kit) (Stool DNA test (mt-sDNA))	Population-based—home (mailing invitation and test kits) Office visit-based—workplace (recruitment) and home (mailing test kits)	USA
Shima et al. [60]	Moderate	Low	Randomized Controlled Trial	Breast	Supermarket Stores	Screening promotion (Educational leaflet) + Screening uptake (Free screening service at workplace and reimbursement) (Mammography)	Workplace (Intervention group) and outside of workplace (Control group)	Japan
Ueda et al. [59]	Moderate	Moderate	Uncontrolled post-test only study	Cervical	Cambodia’s Phnom Penh Special Economic Zone	Screening promotion (Educational sessions) + Screening uptake (Free screening service) (HPV test)	Workplace	Cambodia
Warner et al. [45]	High	Low	Uncontrolled before-after study	Colorectal + Cervical + Breast	Professional home cleaning, hotel cleaning, construction, transportation, and culinary/restaurant service businesses	Screening promotion (Educational session, navigation services) + Screening uptake Free test kits (FIT for colorectal cancer)	Workplace, home, electronic (via phone call) and other locations selected by participants (Financial Incentives)	USA
*Workplace related to health service								

### Characteristics of studies

Among the twenty-one articles included and analysed, twelve papers addressed intervention effectiveness [41–43, 47, 50, 52, 53, 56, 57, 59–61], one article addressed facilitators and barriers for workplace cancer screening interventions [41–43, 47, 50, 52, 53, 56, 57, 59], and eight articles addressed both [48]. Five studies examined breast cancer, six colorectal cancer, four cervical cancer, and one article examined breast and cervical cancers. The remaining four articles discussed all three types (Table 2). There was no study examining workplace lung cancer screening that met the inclusion criteria.

Five studies were randomized controlled trials [42, 49, 57, 58], fifteen were non-randomized trials [41, 43–47, 50–56, 59, 61], and one study used a mixed-method (non-randomized trial and qualitative methods) approach [48]. Eleven articles were from the U.S. (*n* = 11) [41, 43–47, 49, 52–54], and one article each was identified from Japan [60], Turkey [56], Iran [57], Ghana [48], Cambodia [59], Qatar [58], Argentina [55], Singapore [51], China [50] and Malaysia [42].

### Demographic characteristics of participants

The review covered more than 6 million participants (one article did not mention number of participants) [55] (Appendix F). Participants’ age ranged from 18 to 70 + years (Appendix F). In six studies, the sample population consisted of employees and their dependents [41, 44, 46, 47, 53, 55]. Both males and females were invited to colorectal cancer programs while only females were invited to the breast and cervical cancer interventions (except one health education program for health professionals) [51] (Appendix F). Female participant predominance was observed in five interventions, ranging from 56.8% to 71.8% of the study sample [44, 47, 49, 54, 55].

### Characteristics of interventions

Out of the twenty-one included articles, ten articles implemented cancer screening promotion interventions [42, 43, 45, 50–52, 56–58] while fifteen articles offered cancer screening uptake interventions [41, 44–50, 52–56, 59] (Table 2). The interventions were implemented and delivered across a diverse range of workplaces without any clear patterns emerging, including schools, nail salons and manufacturing sites (Table 2). One exception was the healthcare related sector that featured in 5 of the 19 studies [44, 48, 49, 51, 54].

For all cancer types, in addition to covering the costs of screening services, some interventions offered financial incentives to participants (e.g., cash [41, 43, 45] or gift cards [46]) (Table 2). Two interventions used gift draws to increase participation [53, 54], while another provided incentives in the form of rewards tied to activity completion such as health savings, reimbursement, or incentive accounts [44]. Other types of incentives were bonuses, premium reductions, paid time off work [41, 44], and small gifts (e.g. T-shirts, hats, pens, drinks, snacks, household gifts) [48, 53].

In the articles that specified the intervention providers, specialists such as physicians, surgeons, gynaecologists, and nurses were involved in offering educational seminars, health talks and cancer screening services, in addition to the researchers and responsible persons from the workplace [48, 51, 54, 59]. Furthermore, cooperation with lay health workers (trained health workers without formal professional or paraprofessional certificates [62]), health educators and primary health workers was documented [43, 45, 58]. Peer champions (‘peer colon cancer champions’) were used in one workplace; these were volunteers from within the workplace interested in colon cancer often due to personal experience or experience among family and friends [52]. The locations where the interventions took place were either physically in the workplace and/or at clinics, as some workplaces refer or support with navigation to the national cancer screening programmes. A number of interventions were online and implementation was also supported by mailing letters and test kits directly to employees' residences (Table 2) [45–47]. One study examined the effectiveness of allowed paid time off work (paid-sick-leave mandates) in colorectal and breast cancer screening after mandates have been active in some states of the United States [61].

With regard to the type of screening tests, mammography [43, 45, 58] was promoted and used for screening of breast cancer. For colorectal cancer, stool DNA test (mt-sDNA) [47], faecal immunochemical tests (FIT/iFOBT) [45–47], and colonoscopy [61] were utilised. HPV test (self-sampling) [43, 50, 56, 57, 63], HPV test (clinician sampling) [47] and Pap smear test [45, 53–55] were used for cervical cancer. The type of screening tests used was not specified in four articles [49].

### Outcomes

Overall, twenty articles addressed the research question 1 on the effectiveness of workplace interventions (Appendix E). Of these, nine articles included outcomes for changes in knowledge level for breast cancer (*n* = 6), colorectal cancer (*n* = 2), and cervical cancer (*n* = 4), and fifteen articles assessed the screening rate for breast cancer (*n* = 8), colorectal cancer (*n* = 11), and cervical cancer (*n* = 5). In addition, nine articles addressed our second research question (i.e. the faciliators and barriers affecting intervention implementation, delivery and effectiveness); three articles included factors influencing the impact of cancer screening promotion interventions for breast cancer (*n* = 2) and cervical cancer (*n* = 1), and seven articles included factors influencing cancer screening for breast cancer (*n* = 2), colorectal cancer (*n* = 7), and cervical cancer (*n* = 2) (Appendix E).

### Effect direction and magnitude of effectiveness of workplace cancer screening promotion interventions

The workplace cancer screening promotion interventions showed positive impact in all interventions of breast, colorectal and cervical cancer. A number of screening promotion interventions assessed changes in knowledge as well as subsequent changes in screening rate as well (even though the promotion intervention did not offer screening per se), as shown in details in Table 3. The primary study outcomes and original results are available for reference in Appendix I.

**Table 3:** Effect direction plot and magnitude of effectiveness of workplace cancer screening promotion interventions

**Study**	**Study Design**	**Breast Cancer**	**Colorectal Cancer**	**Cervical Cancer**	**ROB**
Abdullah et al. [42]	RCT			▲ (8% difference in screening rate between intervention and control group)	aLow
Callison et al.** [61]	UBAS	▲ (1.22% difference in screening rate at 12 month-rate, 2.07% difference in screening uptake at 24 month-rate)	▲ 1.31% difference in screening rate at 12 month-rate, 1.56% difference in screening uptake at 24 month-rate)		aLow
Eljack et al. [58]	RCT			▲ (50% difference in knowledge construct)	aLow
Esmat Heydari, Azita Noroozi [57]	RCT	▲ (% increase of screening rate – 55% in multimedia education and 80% in group education)			aLow
Fernandez-Esquer et al. [43]	UBAS	▲ (68.7% increase in screening rate)		▲ (66.1% increase in screening rate)	aLow
Hing et al. * [51]	UBAS	▲ (75% improvement in knowledge construct)			aLow
Rafie et al. [52]	UBAS		▲ (40% increase in knowledge construct)		aLow
Warner et al. [45]	UBAS	▲ (14.5% increase in correct age and 5.8% increase in correct frequency)	▲ (12.6% increase in correct age and 12.5% increase in correct frequency)	▲ (30.9% increase in correct age and 12.4% increase in correct frequency)	aLow
Ma et al. [50]	CBAS	▲ (80% improvement in knowledge constructs)			bSomeConcerns
Ozerdogan et al. [56]	UBAS	▲ (mean of 1.32 increase in knowledge level)			bSomeConcerns
*P*-value***		0.0313	0.5	0.125	

Study design: *RCT *Randomised Controlled Trial, *CBAS* Controlled Before-After Study, *UBAS* Uncontrolled Before-After Study, *ROB* Risk of Bias

Effect direction: upward arrow ▲= positive impact, downward arrow ▼= negative impact, sideways arrow ◄►= no change/mixed effects/conflicting findings

Size of arrows: Magnitude of effectiveness of intervention: large arrow ▲ = more than 30% of increase in knowledge/screening rate; medium arrow ▲ = 5- 30% of increase in knowledge/screening rate; small arrow ▲ = <5% of increase in knowledge/screening rate

^*^Workplace related to health service

^**^The study is a modelled estimate based on a sample of population

^***^p-values: from the sign test (two-tailed *p*-value) performed by calculating the number of interventions with positive and negative effect directions [39]

As shown in Table 3, the magnitude of effectiveness for breast cancer screening promotion interventions displayed a 5- 30% increase in knowledge or screening rate in two interventions [45, 56], while four interventions had a greater than 30% increase in knowledge or screening rate on cancer, cancer screening or both [43, 50, 51, 57]. The intervention by Callison et al. predicted a smaller increase with < 5% in screening rate [61].

For colorectal cancer interventions, the study by Rafie et al. showed > 30% increase in knowledge [52]. One intervention displayed a 5- 30% increase in knowledge or screening rate [45, 52], while the final intervention showed < 5% increase in screening rate [61].

For cervical cancer, three out of four interventions had a great than 30% increase in knowledge or screening rate by workplace interventions [43, 45, 58], while one intervention showed 5–30% increase in screening rate [42]. The magnitude (%) change, i.e. increase in knowledge or screening rate for workplace cancer screening knowledge interventions, detailed by study and cancer type can be seen in details in Table 3.

### Effect direction and magnitude of effectiveness of workplace cancer screening uptake interventions

For workplace cancer screening uptake interventions, a positive effect direction was observed for the majority (18/22), while 4 interventions showed no change or mixed effects.

Among breast cancer screening uptake interventions, six interventions showed positive direction (i.e. increase in rate) while one intervention showed mixed results [44], as presented in Table 4. The primary outcomes and original study results are detailed in Appendix J. Out of the six interventions with positive impacts, three interventions had increase in cancer screening rates of > 30% [46, 50, 56], one had > 30% difference between intervention and control groups [60], one had an increase rate of 5–30% [41], and the final intervention showed an increased rate of < 5% [45].

**Table 4:** Effect direction plot and magnitude of effectiveness of workplace cancer screening uptake interventions

Study	Study Design	Breast Cancer	Colorectal Cancer	Cervical Cancer	ROB
Bardach et al. [55]	CBAS		▲ (16 times higher in screening rate)		aLow
Hui et al. [46]	UBAS	▲ (52.4% increase in screening rate)	▲ (33.5% increase in screening rate)	▲ (41.3% increase in screening rate)	aLow
Jensen et al. * [49]	RCT		▲ (Screening rate within sample population—6% in Stock Condition, 16% in Narrative approach, 8.6% in Tailored approach and 15% in Tailored Narrative approach)		aLow
Rafie et al. [52]	UBAS		▲ (20.6% increase in screening rate)		aLow
Shepherd et al. [47]	CPTS		▲ (increase in screening rate—63.2% in Office visit-based and 10.5% in Population-based)		aLow
Shima et al. [60]	RCT	▲ (45.7% difference in screening rate between intervention and control groups)			aLow
Warner et al. [45]	UBAS	▲ (2.1% increase in screening rate)	▲ (increase in screening rate—43.1% in FIT, 0.9% in Sigmoidoscopy and no increase in Colonoscopy)	▲ (2.2% increase in screening rate)	aLow
Bernstein et al. * [44]	UBAS	◄► (comparison with baseline survey)	◄► (comparison with baseline survey)	◄► (comparison with baseline survey)	bSomeConcerns
Cuellar et al. [41]	CBAS	▲ (5% increase in screening rate)	▲ (7% increase in screening rate)	◄►	bSomeConcerns
Hannon et al. * [54]	UPTS		▲ (4.4% increase in screening rate)		bSomeConcerns
Ma et al. [50]	CBAS	▲ (64.4% increase in screening rate)			bSomeConcerns
O'Keefe et al. [53]	UPTS		▲ (Screening rate within sample population—72.1% in UAH and 76% in USA)		bSomeConcerns
Ozerdogan et al. [56]	UBAS	▲ (50.56% screening rate in sample population)			bSomeConcerns
Ueda et al. [59]	UPTS			▲ (19% screening rate in sample population)	bSomeConcerns
*P*-value**		0.0625	0.0039	0.25	

Study design: *RCT* Randomised Controlled Trial, *CBAS* Controlled Before-After Study, *UBAS* Uncontrolled Before-After Study, *ROB* Risk of Bias

Effect direction: upward arrow ▲= positive impact, downward arrow ▼= negative impact, sideways arrow ◄►= no change/mixed effects/conflicting findings

Size of arrows: Magnitude of effectiveness of intervention: large arrow ▲ = more than 30% of increase in screening rate; medium arrow ▲ = 5- 30% of increase in screening rate; small arrow ▲ = <5% of increase in screening rate

^*^Workplace related to health service

^**^p-values: from the sign test (two-tailed *p*-value) performed by calculating the number of interventions with positive and negative effect directions [39]

For colorectal cancer, nine out of ten interventions experienced increases in screening, and one intervention had mixed results [44]. Among these nine interventions, five interventions had > 30% increase in cancer screening rate [45–47, 53, 55], two had 5–30% increase [41, 52], one had 5–30% difference between study groups, and one had < 5% [54].

For cervical cancer screening uptake interventions, three out of five interventions had positive impacts and two interventions had mixed results. Among the interventions with positive impacts, one intervention had a > 30% [46] increase in cancer screening rate, one had 5–30% [59], while the final intervention with a positive impact had a < 5% increase in screening rate [45]. Magnitude (%) of increase in screening can be seen in details in Table 4.

### Factors positively influencing workplace screening promotion and *cancer* screening uptake interventions

A study by Warner et al. found that having health insurance was shown to be associated with improving cancer screening knowledge in breast cancer screening promotion interventions [45] (Appendix G). No factors influencing workplace cancer screening promotion interventions for colorectal and cervical cancer were explored in the included studies.

One factor positively influencing workplace screening uptake interventions reported by the authors of the included studies was living in suburban/urban areas [46]. Gender showed mixed results. In one intervention for colorectal cancer, being male had higher uptake of colorectal cancer screening compared with females, whereas another intervention showed the opposite result with females (particularly aged 50–59 years) having higher uptake than males [45, 55] (Appendix G). Specifically for healthcare personnel, prior use of the healthcare system, interest in health (e.g. having attended previous seminars, greater time spent on the digital health company’s platform and good elaboration on using colorectal cancer screening), and access to primary care, were commonly associated with increased cancer screening rate [44, 49, 51, 54] (Appendix G).

### Factors negatively influencing the workplace *cancer* screening promotion and screening uptake interventions

Factor that negative influence workplace colorectal cancer screening uptake interventions included increased cancer information overload (“a disposition that may be cultivated by communicating cancer information too frequently or in a way that hinders effective processing”) which related to lower uptake of FOBT and colonoscopy [49] (Appendix H). Furthermore, most common barriers to cervical cancer screening uptake included fear of cancer and embarrassment of the procedures during screening tests [58]. Barriers to workplace interventions on breast cancer were not explored in any of the included articles.

## Discussion

This systematic review assessed the effectiveness of workplace cancer screening promotion interventions, including educational sessions, distribution of educational materials, reminder phone calls, navigation to screening services and free time to attend screening, as well as cancer screening uptake interventions that included free screening services, distribution of screening test kits and financial coverage of screening services for the four most common types of cancer. Overall, for both type of interventions for breast, colorectal and cervical cancers, positive impacts were observed on the effect direction plots. For magnitude of effectiveness in both cancer screening promotion and screening uptake interventions, a more than 30% increase in knowledge or cancer screening rate was observed for most breast cancer interventions while the range varied for colorectal and cervical cancer interventions. No studies examining workplace interventions for lung cancer were included, possibly due to its recommendation as a screening programme being directed to high-risk populations. Of note, there were no workplace interventions based in Europe identified in this review.

### Results in the context of previous studies

The cancer screening rates of breast cancer screening uptake interventions in this review differed from an older review of worksite breast cancer screening programs. Caplan et al. (1998) concluded that screening rates increased between 26 to 49% among participants [64]. However, the range of screening found in the current review was broader, ranging from 2.1% to 64.4%. The possible reason for this difference may be due to baseline screening rates observed in the included articles in our review. For instance, in the article by Warner et al., the baseline screening rate of breast cancer was already high at 66.4%, and only experienced an increase of 2.1% after the workplace educational sessions [45].

This current review also found that workplace cancer screening uptake interventions could effectively improve colorectal cancer screening. Similarly, a study of colorectal cancer screening in firefighters organised by the San Francisco Firefighters Cancer Prevention Foundation (SFFCPF) and not by the workplace, concluded that interventions in workplaces could increase the use of screening services (33% increase in FIT usage) [65].

The current review saw low magnitude of effectiveness for cervical cancer screening in four out of five interventions. A similar previous study, implemented in 2009, offering cervical cancer screening through an organised screening program showed that more than half of the participants took the Pap test at least once during the follow-up [66]. This rate is higher than the interventions in our review with the same context.

### Strength and limitations

A strength of this review is that there were no geographical restrictions, providing a global perspective. Furthermore, by not limiting to one type of cancer, this review was able to offer common components and delivery methods employed in workplace interventions addressing cancer screening. However, some limitations should also be noted. The first limitation of this review relates to potential publication bias, as studies with negative or insignificant results are less likely to be published [67]. Secondly, there is a possibility of missing articles. Despite a robust search strategy, this study included published articles in English, and thus has the possibility of missing other relevant articles written in other languages and grey literature sources. A single reviewer undertook most of the screening, but we did have robust measures in place to mitigate any issues (e.g. all papers that were not clearly excluded were discussed between two authors and all final full texts checked by two authors). Another limitation is the lack of comparison of the effect size across interventions, as a meta-analysis was not performed due to the heterogeneity of included studies. Lastly, while incorporating diverse geographical regions and various types of worksites is a strength, it is acknowledged that the effectiveness, facilitators and barriers, may differ due to the working environment and cultural context.

### Implications

Workplace cancer screening interventions can benefit both employees and employers [68–70]. Furthermore, the benefits of screening for common cancers in workplaces can extend to primary and secondary care services as these interventions can lead to early cancer diagnosis. For example, if a 5% increase in colorectal screening rate (60,000 employees) can be achieved in a workplace with over one million employees, then there is a good chance of catching more than 1,000 FOBT-positive tests for colorectal cancer [44, 71]. Similarly, rises in screening uptakes in small and medium-sized workplaces will also collectively increase the possibility of diagnosing cancer cases. This again can lead to reducing health care costs and the workload of already-stretched healthcare services [72].

To implement successful interventions, workplaces are suggested to undergo pilot tests, adapt the educational sessions and screening services according to pre-test surveys, provide multiple education and service-providing sessions, and take into consideration individual preferences for screening (e.g. self-sampling of cervical cancer tests) [44, 46, 51, 55]. Moreover, workplaces are suggested to promote cancer screening by allowing employees to attend cancer screening during working time [73].

Care must be taken to ensure that inequalities in cancer screening are addressed but also that they are not exacerbated (e.g., between employed and unemployed populations, but also between employees). Workplaces need to ensure that screening interventions are available to all employees and barriers to specific employee groups are minimised. Researchers, the health service and workplaces should cooperate to offer health programs to hard-to-reach professions and blue-collar workers to prevent exacerbating inequalities [74, 75]. To minimise inequality between employed and unemployed populations, employers can extend their interventions to employees’ families and dependents wherever possible [76].

This review found that barriers to participating in workplace cancer screening promotion programs and utilising cancer screening services were less explored. Therefore, researchers and worksites should find novel ways to discover these factors as they are vital in improving the efficacy of workplace interventions. Additionally, utilizing cervical cancer screening services in workplace interventions showed a low magnitude of effectiveness. Future research should explore the reasons behind this in order to effectively improve screening uptake. In addition, workplaces should ensure that screening services are offered and tested with informed choice on possible results and potential harms.

Furthermore, longitudinal studies with larger samples and studies with long-term follow-up should be carried out to explore the effectiveness of these interventions and the potential impact these interventions may have, not only on screening uptake but on detection, treatment options, and ultimately survival rates, as well as on non-cancer outcomes including employment and mental health. Rigorous evaluations of the interventions are also necessary to identify the impact on cancer screening uptake and how workplace screening could be linked to any organised screening programmes to avoid duplication.

## Conclusion

Cancer is one of the leading causes of death globally [77], and its rising number of new cases every year has been alarming, calling for urgent action on prevention and early detection of the disease. This systematic review presents a comprehensive overview examining the effectiveness and factors influencing workplace interventions in promoting screening and increasing cancer screening uptake in employees.

Our findings suggest that workplace interventions can have a positive impact in promoting cancer screening, increasing knowledge around screening and on cancer screening rates that may results in the earlier detection of breast, colorectal and cervical cancers in working age adults. Our findings also suggest that workplaces and employers can design evidence-based, structured and effective interventions by working side by side with researchers, public health specialists and health care systems [50, 55, 78]. Within the workplace, human and material resources would need to be allocated to make such interventions sustainable and involvement should be shown all stakeholders to create workplaces that promote health [52]. Our findings also conveyed that factors that may impede participation in workplace programs should be examined in further research as very few studies have focused on these.

In the past 3 years, while cancer cases were expected to be rising, public and healthcare personnel’s attentions were focused on the Covid-19 pandemic by redirecting material and human resources to the pandemic and restricting non-urgent healthcare services. Now more than ever, it is imperative to take action to combat the global burden of cancer. Evidence-informed, rigorous approaches and novel settings such as workplaces can be used to tackle the burden of cancer and improve health not only for employees but for the wider public as well.

### Supplementary Information

Supplementary Material 1.

## Data Availability

This systematic review collected the data from published articles and all the data extracted are included in the supplementary material.

## Abbreviations

ARBA: Internal Revenue Agency of the Province of Buenos Aires
BSE: Breast Self-examination
CASP: Critical Appraisal Skills Programme
CBE: Clinical Breast Examination
CINAHL: Cumulative Index to Nursing and Allied Health Literature
CONSORT: Consolidated Standards of Reporting Trials
FIT: Faecal Immunochemical Test
FOBT: Faecal Occult Blood Test
HPV: Human Papillomavirus
hrHPV: High-risk Human Papillomavirus
iFOBT: Immunochemical fecal occult blood test
mt-sDNA: Multi-target stool DNA
NHS: National Health Service
PSA: Prostate-specific Antigen
ROB2: Revised version of Cochrane Risk of Bias tool
ROBIN-I: Risk Of Bias In Non-randomised Studies of Interventions
TREND: Transparent Reporting of Evaluations with Nonrandomized Design
UAH: University of Alabama in Huntsville
UK: United Kingdom
U.S.: United States
USA: University of South Alabama
USPSTF: United States Preventive Services Task Force
