# Supplementary material for: Effectiveness of workplace cancer screening interventions: a systematic review
Source: BMC Cancer. 2024 Aug 12;24:999. doi: 10.1186/s12885-024-12649-0 (PMC11321184; doi:10.1186/s12885-024-12649-0)
Supplement: Supplementary file 1 — Supplementary Material 1. [file 12885_2024_12649_MOESM1_ESM.docx]

**Effectiveness of Workplace Cancer Screening Interventions:**

**A Systematic Review**

***[Supplementary Material]***

TABLE OF CONTENTS

[Appendix A. Cancer screening recommendations for five common cancer types by the United States Preventive Services Task Force 2](#_Toc171283744)

[Appendix B. Search strategy in remaining databases 3](#_Toc171283745)

[Appendix C. Quality assessment of included studies 5](#_Toc171283746)

[Appendix D.1. Risk of bias assessment of non-randomized trials 6](#_Toc171283747)

[Appendix D.2. Risk of bias assessment of randomized controlled trials 7](#_Toc171283748)

[Appendix E. Flow diagram of subgrouping in narrative analysis 8](#_Toc171283749)

[Appendix F. Demographic characteristics of participants 9](#_Toc171283750)

[Appendix G. Factors positively influencing workplace cancer screening promotion and screening uptake interventions 15](#_Toc171283751)

[Appendix H. Factors negatively influencing workplace cancer screening promotion and screening uptake interventions 17](#_Toc171283752)

[Appendix I. Effectiveness of workplace cancer screening promotion interventions (Results as in original articles) 18](#_Toc171283753)

[Appendix J. Effectiveness of workplace cancer screening uptake interventions (Results as in original articles) 20](#_Toc171283754)

[Appendix K. Studies excluded from the analysis (Results as in original studies) 23](#_Toc171283755)

## Appendix A. Cancer screening recommendations for five common cancer types by the United States Preventive Services Task Force

| **Type of cancer** | **Recommended tests and frequency** | **Population** |
| --- | --- | --- |
| Breast [1, 2] | - Mammography once every 2 years (Biennial) | - Women aged 40 to 74 years. (Grade B recommendation) |
| Prostate [3] | - Periodic prostate-specific antigen (PSA)–based screening | - Men aged 55 to 69 years (Grade C recommendation - individual decision) - Men 70 years and older (Grade D recommendation) |
| Lung [4] | - Low dose computed tomography (CT) every year | - Adults aged 50 to 80 years who have a 20-pack-year smoking history and currently smoking or have quit within the past 15 years (Grade B recommendation) - **Note:** Screening should be discontinued once a person has not smoked for 15 years or develops a health problem that substantially limits life expectancy or the ability or willingness to have curative lung surgery. |
| Colorectum [5] | - High-sensitivity guaiac faecal occult blood test (HSgFOBT) or faecal immunochemical test (FIT) every year - Stool DNA-FIT every 1 to 3 years - Computed tomography colonography every 5 years - Flexible sigmoidoscopy every 5 years - Flexible sigmoidoscopy every 10 years + annual FIT - Colonoscopy screening every 10 years | - Adults aged 50 to 75 years (Grade A recommendation) - Adults aged 45 to 49 years (Grade B recommendation) - Adults aged 76 to 85 years (Grade C recommendation – Selective screening considering the patient’s overall health, prior screening history, and patient’s preferences.) |
| Cervix uteri [6] | - Cervical cytology along every 3 years - Cervical cytology alone every 3 years, high-risk human papillomavirus (hrHPV) testing alone every 5 years, or hrHPV testing in combination with cytology every 5 years - Not recommended for screening (<21 years old, >65 years old) | - Women aged 21 to 29 years (Grade A recommendation) - Women aged 30 to 65 years (Grade A recommendation) - Women younger than 21 years, women older than 65 years who have had adequate prior screening and are not otherwise at high risk for cervical cancer, women who have had a hysterectomy with removal of the cervix and do not have a history of a high-grade precancerous lesion or cervical cancer (Grade D recommendation) |

Note: Suggestions for practice by the USPSTF:

Grade A recommendation - Offer or provide this service

Grade B recommendation - Offer or provide this service

Grade C recommendation - Offer or provide this service for selected patients depending on individual circumstances

Grade D recommendation - Discourage the use of this service

## Appendix B. Search strategy in remaining databases

| **No.** | **Database** | **Search strategy** |
| --- | --- | --- |
| 1 | Medline (Ovid) | ((((work* OR job* OR employ* OR occupation*) adj3 (health* OR effect* OR program* OR promot* OR intervention* OR diagnos* OR screen* OR campaign* OR polic* OR service* OR initiative* OR "cancer awareness" OR scan*)).ti,ab OR occupational health [mesh] OR occupational health services [mesh] OR occupational medicine [mesh]) AND ((cancer* OR malignan* OR neoplas* OR tumo$r*).ti,ab OR early detection of cancer [mesh] OR neoplasm [mesh]) AND ((breast* OR lung* OR colorect* OR colon OR rect* OR bowel OR intestinal OR cervi* OR colonoscop* OR sigmoidoscop* OR "computed tomography colonograph*" OR "f$ecal occult blood test*" OR "f$ecal immunochemical test*" OR "stool DNA test*" OR "stool test*" OR "double contrast barium enema" OR mammogra* OR "breast exam*" OR "breast self-exam*" OR "low dose computed tomograph*" OR "chest X-ray*" OR "cervical cytolog*" OR "pap test*" OR "pap smear" OR "high-risk human papillomavirus*" OR hpv* OR hrhpv* ).ti,ab)) AND Limited to publication year – 2010 to the date of search (19th May 2022), English language, research on Human |
| 2 | Cochrane | ((((work* OR job* OR employ* OR occupation*) NEAR/3 (health* OR effect* OR program* OR promot* OR intervention* OR diagnos* OR screen* OR campaign* OR polic* OR service* OR initiative* OR "cancer awareness" OR scan*)).ti,ab OR occupational health [mesh] OR occupational health services [mesh] OR occupational medicine [mesh]) AND ((cancer* OR malignan* OR neoplas* OR tumo$r*).ti,ab OR early detection of cancer [mesh] OR neoplasms [mesh]) AND ((breast* OR lung* OR colorect* OR colon OR rect* OR bowel OR intestinal OR cervi* OR colonoscop* OR sigmoidoscop* OR "computed tomography colonograph*" OR "f$ecal occult blood test*" OR "f$ecal immunochemical test*" OR "stool DNA test*" OR "stool test*" OR "double contrast barium enema" OR mammogra* OR "breast exam*" OR "breast self-exam*" OR "low dose computed tomograph*" OR "chest X-ray*" OR "cervical cytolog*" OR "pap test*" OR "pap smear" OR "high-risk human papillomavirus*" OR hpv* OR hrhpv* ).ti,ab)) AND Limited to publication year – 2010 to the date of search (19th May 2022) |
| 3 | Web of Science (Core collection) | ((((work* NEAR/3 health*) OR (job* NEAR/3 health*) OR (employ* NEAR/3 health*) OR (occupation* NEAR/3 health*) OR (work* NEAR/3 effect*) OR (job* NEAR/3 effect*) OR (employ* NEAR/3 effect*) OR (occupation* NEAR/3 effect*) OR (work* NEAR/3 "cancer awareness") OR (job* NEAR/3 "cancer awareness") OR (employ* NEAR/3 "cancer awareness") OR (occupation* NEAR/3 "cancer awareness") OR (work* NEAR/3 initiative*) OR (job* NEAR/3 initiative*) OR (employ* NEAR/3 initiative*) OR (occupation* NEAR/3 initiative*) OR (work* NEAR/3 service*) OR (job* NEAR/3 service*) OR (employ* NEAR/3 service*) OR (occupation* NEAR/3 service*) OR (work* NEAR/3 polic*) OR (job* NEAR/3 polic*) OR (employ* NEAR/3 polic*) OR (occupation* NEAR/3 polic*) OR (work* NEAR/3 campaign*) OR (job* NEAR/3 campaign*) OR (employ* NEAR/3 campaign*) OR (occupation* NEAR/3 campaign*) OR (work* NEAR/3 screen*) OR (job* NEAR/3 screen*) OR (employ* NEAR/3 screen*) OR (occupation* NEAR/3 screen*) OR (work* NEAR/3 diagnos*) OR (job* NEAR/3 diagnos*) OR (employ* NEAR/3 diagnos*) OR (occupation* NEAR/3 diagnos*) OR (work* NEAR/3 intervention*) OR (job* NEAR/3 intervention*) OR (employ* NEAR/3 intervention*) OR (occupation* NEAR/3 intervention*) OR (work* NEAR/3 promot*) OR (job* NEAR/3 promot*) OR (employ* NEAR/3 promot*) OR (occupation* NEAR/3 promot*) OR (work* NEAR/3 program*) OR (job* NEAR/3 program*) OR (employ* NEAR/3 program*) OR (occupation* NEAR/3 program*) OR (work* NEAR/3 scan*) OR (job* NEAR/3 scan*) OR (employ* NEAR/3 scan*) OR (occupation* NEAR/3 scan*)).ti,ab) AND ((cancer* OR malignan* OR neoplas* OR tumo$r*).ti,ab) AND ((breast* OR lung* OR colorect* OR colon OR rect* OR bowel OR intestinal OR cervi* OR colonoscop* OR sigmoidoscop* OR "computed tomography colonograph*" OR "f$ecal occult blood test*" OR "f$ecal immunochemical test*" OR "stool DNA test*" OR "stool test*" OR "double contrast barium enema" OR mammogra* OR "breast exam*" OR "breast self-exam*" OR "low dose computed tomograph*" OR "chest X-ray*" OR "cervical cytolog*" OR "pap test*" OR "pap smear" OR "high-risk human papillomavirus*" OR hpv* OR hrhpv* ).ti,ab)) AND Limited to publication year – 2010 to the date of search (19th May 2022), English language, research on Human, and excluding the research areas not related to the research topic |
| 4 | Scopus | ((((work* W/3 health*) OR (job* W/3 health*) OR (employ* W/3 health*) OR (occupation* W/3 health*) OR (work* W/3 effect*) OR (job* W/3 effect*) OR (employ* W/3 effect*) OR (occupation* W/3 effect*) OR (work* W/3 "cancer awareness") OR (job* W/3 "cancer awareness") OR (employ* W/3 "cancer awareness") OR (occupation* W/3 "cancer awareness") OR (work* W/3 initiative*) OR (job* W/3 initiative*) OR (employ* W/3 initiative*) OR (occupation* W/3 initiative*) OR (work* W/3 service*) OR (job* W/3 service*) OR (employ* W/3 service*) OR (occupation* W/3 service*) OR (work* W/3 polic*) OR (job* W/3 polic*) OR (employ* W/3 polic*) OR (occupation* W/3 polic*) OR (work* W/3 campaign*) OR (job* W/3 campaign*) OR (employ* W/3 campaign*) OR (occupation* W/3 campaign*) OR (work* W/3 screen*) OR (job* W/3 screen*) OR (employ* W/3 screen*) OR (occupation* W/3 screen*) OR (work* W/3 diagnos*) OR (job* W/3 diagnos*) OR (employ* W/3 diagnos*) OR (occupation* W/3 diagnos*) OR (work* W/3 intervention*) OR (job* W/3 intervention*) OR (employ* W/3 intervention*) OR (occupation* W/3 intervention*) OR (work* W/3 promot*) OR (job* W/3 promot*) OR (employ* W/3 promot*) OR (occupation* W/3 promot*) OR (work* W/3 program*) OR (job* W/3 program*) OR (employ* W/3 program*) OR (occupation* W/3 program*) OR (work* W/3 scan*) OR (job* W/3 scan*) OR (employ* W/3 scan*) OR (occupation* W/3 scan*)).ti,ab) AND ((cancer* OR malignan* OR neoplas* OR tumo$r*).ti,ab) AND ((breast* OR lung* OR colorect* OR colon OR rect* OR bowel OR intestinal OR cervi* OR colonoscop* OR sigmoidoscop* OR "computed tomography colonograph*" OR "f$ecal occult blood test*" OR "f$ecal immunochemical test*" OR "stool DNA test*" OR "stool test*" OR "double contrast barium enema" OR mammogra* OR "breast exam*" OR "breast self-exam*" OR "low dose computed tomograph*" OR "chest X-ray*" OR "cervical cytolog*" OR "pap test*" OR "pap smear" OR "high-risk human papillomavirus*" OR hpv* OR hrhpv* ).ti,ab)) AND Limited to publication year – 2010 to the date of search (19th May 2022), English language, and excluding the research areas not related to the research topic |
| 5 | CINAHL | ((((work* OR job* OR employ* OR occupation*) N3 (health* OR effect* OR program* OR promot* OR intervention* OR diagnos* OR screen* OR campaign* OR polic* OR service* OR initiative* OR "cancer awareness" OR scan*)).ti,ab OR occupational health services [mesh]) AND ((cancer* OR malignan* OR neoplas* OR tumo$r*).ti,ab OR neoplasm metastasis [mesh] OR early detection of cancer [mesh]) AND ((breast* OR lung* OR colorect* OR colon OR rect* OR bowel OR intestinal OR cervi* OR colonoscop* OR sigmoidoscop* OR "computed tomography colonograph*" OR "f$ecal occult blood test*" OR "f$ecal immunochemical test*" OR "stool DNA test*" OR "stool test*" OR "double contrast barium enema" OR mammogra* OR "breast exam*" OR "breast self-exam*" OR "low dose computed tomograph*" OR "chest X-ray*" OR "cervical cytolog*" OR "pap test*" OR "pap smear" OR "high-risk human papillomavirus*" OR hpv* OR hrhpv* ).ti,ab)) AND Limited to publication year – 2010 to the date of search (19th May 2022) and English language |

## Appendix C. Quality assessment of included studies

| **Study ID** | **Percentage achieved** | **Result of quality assessment** |  | **Study ID** | **Percentage achieved** | **Result of quality assessment** |
| --- | --- | --- | --- | --- | --- | --- |
| Abdullah et al [7] | 82% | High |  | Hui et al [8] | 82% | High |
| Ahmed et al [9] | 43% | Low |  | Jensen et al * [10] | 74% | Moderate |
| Bardach et al [11] | 77% | Moderate |  | Kim et al [12] | 55% | Low |
| Behnke et al * [13] | 82% | High |  | Ma et al [14] | 77% | Moderate |
| Bernstein et al * [15] | 82% | High |  | Matsuura et al [16] | 41% | Low |
| Callison et al [17] | 68% | Moderate |  | McFall et al [18] | 48% | Low |
| Cuellar et al [19] | 82% | High |  | O'Keefe et al [20] | 66% | Moderate |
| Eljack et al [21] | 62% | Moderate |  | Ozerdogan et al [22] | 66% | Moderate |
| Esmat Heydari, Azita Noroozi [23] | 60% | Moderate |  | Rafie et al [24] | 61% | Moderate |
| Fernandez-Esquer et al [25] | 82% | High |  | Shepherd et al [26] | 82% | High |
| Greenwald et al [27] | 61% | Moderate |  | Shima et al [28] | 72% | Moderate |
| Hafeez et al [29] | 41% | Low |  | Ueda et al [30] | 61% | Moderate |
| Hannon et al * [31] | 73% | Moderate |  | Warner et al [32] | 84% | High |
| Hing et al * [33] | 64% | Moderate |  |  |  |  |

*Workplace related to health service

| Appendix D.1. Risk of bias assessment of non-randomized trials | | | | | | | | |
| --- | --- | --- | --- | --- | --- | --- | --- | --- |
| **Study ID** | **Bias - confounding** | **Bias - participant selection** | **Bias -classification of intervention** | **Bias -fidelity of intervention** | **Bias - missing data** | **Bias -measure-ment of outcomes** | **Bias -selection of reported result** | **Overall Risk of Bias (RoB)** |
| Ahmed et al [9] | Low | Low | Low | Low | Moderate | Low | Low | Moderate |
| Bardach et al [11] | Low | Low | Low | Low | Low | Low | Low | Low |
| Behnke et al * [13] | Low | Low | Low | Low | Low | Low | Low | Low |
| Bernstein et al * [15] | Moderate | Low | Low | Low | Low | Low | Low | Moderate |
| Callison et al [17] | Low | Low | Low | Low | Low | Low | Low | Low |
| Cuellar et al [19] | Moderate | Low | Low | Low | Low | Low | Low | Moderate |
| Fernandez-Esquer et al [25] | Low | Low | Low | Low | Low | Low | Low | Low |
| Greenwald et al [27] | Moderate | Serious | Moderate | Low | Low | Low | Low | Serious |
| Hafeez et al [29] | Low | Moderate | Low | Moderate | Moderate | Low | Low | Serious |
| Hannon et al * [31] | Moderate | Low | Low | Low | Low | Low | Low | Moderate |
| Hing et al * [33] | Low | Low | Low | Low | Low | Low | Low | Low |
| Hui et al [8] | Low | Low | Low | Low | Low | Low | Low | Low |
| Kim et al [12] | Moderate | Low | Low | Low | Low | Low | Low | Moderate |
| Ma et al [14] | Moderate | Low | Low | Low | Low | Low | Low | Moderate |
| Matsuura et al [16] | Moderate | Moderate | Low | Low | Low | Moderate | Low | Serious |
| McFall et al [18] | Moderate | Moderate | Low | Low | Moderate | Low | Low | Serious |
| O'Keefe et al [20] | Moderate | Low | Low | Low | Low | Low | Low | Moderate |
| Ozerdogan et al [22] | Moderate | Low | Low | Low | Low | Low | Low | Moderate |
| Rafie et al [24] | Low | Low | Low | Low | Low | Low | Low | Low |
| Shepherd et al [26] | Low | Low | Low | Low | Low | Low | Low | Low |
| Ueda et al [30] | Moderate | Low | Low | Low | Low | Low | Low | Moderate |
| Warner et al [32] | Low | Low | Low | Low | Low | Low | Low | Low |
| Behnke et al * [13] | | Assessed using CASP for qualitative approach | | | | | | Low |

*Workplace related to health service

## Appendix D.2. Risk of bias assessment of randomized controlled trials

| **Risk of bias assessment using ROB 2 (cluster randomized trials)** | | | | | | | | |
| --- | --- | --- | --- | --- | --- | --- | --- | --- |
| **Study ID** | **Bias - confounding** | **Bias - participant selection** | **Bias -classification of intervention** | **Bias -fidelity of intervention** | **Bias - missing data** | **Bias -measure-ment of outcomes** | **Bias -selection of reported result** | **Overall Risk of Bias (RoB)** |
| Abdullah et al [7] | Low | Low | Low | NA | Low | Low | Low | Low |
| Eljack et al [21] | Low | Low | NA | Low | Low | Low | Low | Low |
| Shima et al [28] | Low | Low | Low | Low | Low | Low | Low | Low |

| **Risk of bias assessment using ROB 2 (individually randomized trials)** | | | | | | |
| --- | --- | --- | --- | --- | --- | --- |
| **Study ID** | **Risk of bias arising from the randomization process** | **Risk of bias due to deviations from the intended interventions** | **Risk of bias due to missing outcome data** | **Risk of bias in measurement of the outcome** | **Risk of bias in selection of the reported result** | **Overall risk of bias assessment** |
| Jensen et al * [10] | Low | Low | Low | Low | Low | Low |
| Esmat Heydari, Azita Noroozi [23] | Low | Low | Low | Low | Low | Low |

*Workplace related to health service

## Appendix E. Flow diagram of subgrouping in narrative analysis

**21 Articles**

Research Question 2

**9 articles**

Research Question 1

**20 articles**

Outcome:

Cancer Screening Knowledge

**9 articles**

Outcome:

Cancer screening rate

**15 articles**

Outcome:

Cancer screening rate

**7 articles**

Outcome:

Cancer Screening Knowledge

**3 articles**

Breast cancer - **8** **articles**

Colorectal cancer - **11 articles**

Cervical cancer - **5 articles**

Breast cancer - **6 articles**

Colorectal cancer - **2 articles**

Cervical cancer - **4 articles**

Breast cancer - **2 articles**

Colorectal cancer - **7 articles**

Cervical cancer - **2 articles**

Breast cancer - **2 articles**

Colorectal cancer - **0 articles**

Cervical cancer - **1 articles**

| Appendix F. Demographic characteristics of participants | | | | | |
| --- | --- | --- | --- | --- | --- |
| **Study ID** | **Type of cancer** | **Sample size** | **Age** | **Sex** | **Inclusion and exclusion criteria in sample selection** |
| Abdullah et al [7] | Cervical | 403 | Mean age ±SD:  Study group  = 36.1±8.0  Control group  = 36.5±7.3 | Female = 100% | Inclusion criteria: female secondary teachers who were either naive to Pap smear or had their last test more than three years previously |
| Bardach et al [11] | Colorectal | Not specified. | Intervention group:  < 60 years = 86.1% | Intervention group:  Male = 43.2% Female = 56.8% | Inclusion criteria: 50-75 years who were not tested in the last year for each month |
| Behnke et al * [13] (Qualitative method) | Cervical | 10 | Mean age = 41 years Range: 28-59 years | Female = 100% | Inclusion criteria: female healthcare providers having direct contact with patients, and having different professional profiles, training, and age |
| Bernstein et al * [15] | Breast + Colorectal+ Cervical | 1.24 million | Mean age = 45 years | Male = 41% Female = 59% | Inclusion criteria: eligible for incentives through health savings accounts, health reimbursement accounts, health incentive accounts, gift cards, and other means |
| Callison et al [17] | Breast + Colorectal | 1.3 million person-specific records per year for our mammography sample  2.5 million person-specific records per year for our colorectal-cancer screening | Mammography Sample (Mean age)  Madate exposure - 52.3  No Mandate exposure - 52.4  Colorectal Cancer Screening Sample (Mean age)  Mandate Exposure - 54.1  No Mandate Exposure - 54.2 | Breast - Female (100%)  Colorectal cancer screening sample  - Female 48% and Male 52% in mandate exposure  - Female 46% and Male 54% in non-mandate exposure | Inclusion criteria: Breast Cancer - women between 40 and 64 years of age, Workers with continuous plan enrollment for either 12 or 24 months  Inclusion criteria: Colorectal Cancer - adults between 45 and 64 years of age, Workers with continuous plan enrollment for either 12 or 24 months |
| Cuellar et al [19] | Breast + Colorectal+ Cervical | Breast cancer  - 253,632  Cervical cancer  - 366,659  Colorectal cancer  - 233,935 | Range: 18 - 64 years | Intervention group:  Male = 50.1% Female = 49.9%  Control group: Male = 50.7% Female = 49.3% | Inclusion criteria: employees who were covered by the insurer administering the wellness programs for 1 full plan year |
| Eljack et al [21] | Cervical | 432 | Mean age ±SD:  Study 1 = 39.0 +/- 7.1, Control 1 = 38.4 +/- 6.8,  Study 2 = 37.5 +/- 8.4,  Control 2 = 40.0 +/- 5.7 | Female = 100% | Inclusion criteria: according to age, marital status and acceptance to participate in the study  Exclusion criteria: females with previous history of cervical cancer, hysterectomy, or who has been treated for precancerous lesions related to cervical |
| Esmat Heydari,  Azita Noroozi [23] | Breast | 130 | Mean age:  Multimedia education  = 44.92±4.34,   Group education  = 45.48±4.69 | Female = 100% | Inclusion criteria: > 40 years, not pregnant or breast-feeding, not having breast cancer or other types of cancers, not having family history of breast cancer, not having breast biopsy experience and mammography in the past three years |
| Fernandez-Esquer et al [25] | Breast + Cervical | 186 | Mean age: 46.7 years | Female= 100% | Inclusion criteria: (1) ≥ 18 years, (2) self-identified as Vietnamese, (3) lived in the Houston, Texas area for at least three years, and (4) currently working in a nail salon in Houston’s “Asia town” neighborhood, a seven ZIP-code neighborhood with a dense population of Asian businesses and residences |
| Hannon et al * [31] | Colorectal | Educational seminars   - 275   FIT distribution   - 118   Employee survey  - 503 | 39% = 50 years or older | Male = 39% Female = 61% | Inclusion criteria for worksites: (a) located in Spokane County and employed primarily Spokane County residents, (b) were willing to share de-identified data from the Spokane Regional Health District health risk assessment, and (c) were interested in receiving the program  Inclusion criteria for employees: ≥ 50 years, were not currently screened by any method |
| Hing et al * [33] | Breast | 141 | < 40 years = 62.4%  40–49 years = 19.1% ≥ 50 years = 18.4% | Male = 2.1% Female = 97.9% | Inclusion criteria: all staff of the Changi General Hospital |
| Hui et al [8] | Breast + Colorectal+ Cervical | Breast cancer – 210  Cervical cancer – 320  Colorectal cancer – 695 | Breast cancer: 40-49 years = 65.2%  50-59 years = 30.5% ≥60 years = 4.3%  Cervical cancer: 20-29 years = 6.6% 30-39years = 11.6% 40-49 years = 31.9% 50-59 years = 44.7% ≥ 60 years = 5.3%  Colorectal cancer:  50-59 years = 84.6% ≥ 60 years = 15.4% | Both. Not specified in detail. | Inclusion criteria: Kansas state employees and their dependents, enrolled in the Kansas state employee health plan, completed a standard health risk assessment in 2008 and 2009 |
| Jensen et al * [10] | Colorectal | 288 | No specified | Male = 29.2% Female = 71.8% | Inclusion criteria: employees, 50-75 years, behind on their colorectal cancer screening |
| Ma et al [14] | Breast | 453 | Mean age:  Intervention group  = 45.3 years   Control group  = 46.1 years | Female = 100% | Inclusion criteria: ≥ 40 years, no current diagnosis of breast cancer and not having participated in breast cancer screening within the past 12 months |
| O'Keefe et al [20] | Colorectal | University of Alabama in Huntsville (UAH) - 86  University of South Alabama (USA) - 146 | No specified | Both. Not specified in detail. | UAH: Inclusion criteria: ≥ 50 years with no personal or family history of colorectal cancer or precancerous polyps, no history of inflammatory bowel disease, and no history of genetic syndromes  USA: Inclusion criteria: aged 50 years and older |
| Ozerdogan et al [22] | Breast | Educational program  - 903  Breast cancer screening  - 89 | 30-39 years = 64.4%  40-59 years = 35.6% | Female = 100% | Inclusion criteria for educational program:  ≥ 30 years and were literate   Inclusion criteria for cancer screening: attended education program, ≥ 40, and had not had mammography in the last 2 years  Exclusion criteria for cancer screening: women who received breast cancer treatment |
| Rafie et al [24] | Colorectal | 873 | No specified | Not specified | Not specified |
| Shima et al [28] | Breast | 1939 | Intervention Group  Mean age = 52.5  Control Group  Mean age = 53.3 | Female (100%) | Inclusion criteria: Female employees who were at least 40 years old and worked in one of the 25 stores. |
| Shepherd et al [26] | Colorectal | Population-based group - 840  Office visit-based group - 68 | Mean age:  Population-based  = 56.0 (±4.4)  Office visit-based = 56.0 (±4.5) | Population based: Male = 41.8%  Female = 59.2%   Office visit-based: Male = 38% Female = 62% | Inclusion criteria: ≥ 50, identified as due for colorectal cancer screening, and certificated employees and their dependents who are covered by the Metropolitan Nashville Public Schools health plan (population-based outreach)  Exclusion criteria: current evidence or history of being above average- or high-risk for colorectal cancer, losing health plan coverage during the study period or if the initially placed screening test order was canceled prior to shipping, non-certificated employees, ≥ 65years or had Medicare as the primary insurer (population-based outreach) |
| Ueda et al [30] | Cervical | Education session – 2597  Screening – 681 | 15–19 years = 492  20–24 years = 1025  25−29 years = 588 30–39 years = 347 40–49 years = 18  50–59 years = 2 Unknown = 1734 | Female = 100% | Inclusion criteria: > 25 years, ever married, with at least one sexual partner, fully attended the health education program, and not known to be pregnant at the time of testing |
| Warner et al [32] | Breast +  Colorectal +  Cervical | 318 | Completed:  18–30 years = 7.2%  31–49 years = 49.1%  50 and older = 42.6%  Incomplete  18–30 years = 9.4%  31–49 years = 69.8%  50 and older = 20.8% | Both.  Not specified in detail. | Inclusion criteria: Latinos speaking Spanish or English, ≥ 18 years or, and were employed in a service or manual labor job  Inclusion criteria for FIT tests - ≥ 50 years, overdue for colorectal cancer screening |

| Appendix G. Factors positively influencing workplace cancer screening promotion and screening uptake interventions | | | | | |
| --- | --- | --- | --- | --- | --- |
| **Study ID** | **Quality of studies** | **Impact on workplace cancer screening uptake interventions** | | | **Impact on workplace cancer screening promotion interventions** |
|  |  | **Breast Cancer** | **Colorectal Cancer** | **Cervical Cancer** | **Breast Cancer** |
| Behnke et al * [13] | High |  |  | - Fear of cervical cancer - Self-sampling method - Free of cost - Retiring soon |  |
| Bernstein et al * [15] | High | - Having one’s blood pressure checked in the last year - Access to primary care - Greater time spent on the digital health company’s platform | - Having one’s blood pressure checked in the last year - Access to primary care - Greater time spent on the digital health company’s platform | - Having one’s blood pressure checked in the last year - Access to primary care - Greater time spent on the digital health company’s platform |  |
| Hui et al [8] | High | - Residential area (suburban/urban) | - Residential area (suburban/urban) - 2 or more doctor’s visits during  the previous year |  |  |
| Warner et al [32] | High |  | - Sex (Male) - Income (High) |  | - Health Insurance (Having insurance) |
| Bardach et al [11] | Moderate |  | - Sex (Female, particularly aged 50 to 59 years) |  |  |
| Hannon et al * [31] | Moderate |  | - Having attended educational seminars |  |  |
| Hing et al * [33] | Moderate |  |  |  | - Age (women ≥40 years) - Income (High) - Type of dwellings (larger dwellings) - Attending previous talks - >10 years tenure in healthcare - Personal encounter with breast cancer patients |
| Jensen et al * [10] | Moderate |  | - Past screening and good elaboration on using colorectal cancer screening |  |  |
| *Workplace Related to Health service | | | | | |

| Appendix H. Factors negatively influencing workplace cancer screening promotion and screening uptake interventions | | | | | | |
| --- | --- | --- | --- | --- | --- | --- |
| **Study ID** | **Quality of studies** | **Type of cancer** | | **Type of intervention** | **Factors** | |
| Eljack et al [21] | Moderate | Cervical | | Cancer screening promotion intervention | - Fear of cancer/cancer phobia (most common) - Embarrassment (most common) - Difficulty in reaching to screening services - Difficulty in getting appointments - Overcrowding - Lack of knowledge - Lack of time - Feeling of not needing to screen - Refusal - Cost (less common) - Husband refusal (less common) - Traditions/Culture (less common) | |
| Jensen et al * [10] | Moderate | Colorectal | | Cancer screening uptake intervention | - Greater cancer information overload in case of FOBT and colonoscopy | |
| *Workplace Related to Health service | | |  | | |  |

## Appendix I. Effectiveness of workplace cancer screening promotion interventions (Results as in original articles)

| **Study** | **Quality Score** | **Changes in Knowledge or**  **Screening Uptake** | **Results** | **Statistical Results** |
| --- | --- | --- | --- | --- |
| Abdullah et al [7] | High | Screening uptake | *Cervical Cancer Screening (after 24 weeks of follow up),*  Intervention Group – 18.1% ; Control Group – 10.1% | p < 0.05 |
|  |  |  | Pap smear uptake twice as high in intervention compared to control group. | Adjusted OR: 2.44 (95% CI: 1.29–4.62). |
| Fernandez-Esquer et al [25] | High | Screening uptake | Pap test screening uptake significantly associated with intervention (i.e. navigation services) acceptance, compared to those that did not accept. | χ2 = 8.54,  df = 1, p = 0.003 |
|  |  |  | Mammography screening among participants receiving the intervention and those that did not, was not significant (71.4% vs 77.3%). | χ2 = 0.10,  df = 1, p = 0.753 |
| Warner et al [32] | High | Knowledge | *Knowledge of the age at which to begin cancer screenings*   - Cervical cancer - 65.1% baseline vs. 77.7% follow-up - Breast cancer - 67.2% baseline vs. 81.7% follow-up - Colorectal cancer - 49.8% baseline vs. 80.7% follow-up | *Cervical* (p < 0.001),  *Breast (*p < 0.001),  *Colorectal* (p = 0.001) |
|  |  |  | *Knowledge of the frequency of cancer screenings*   - Cervical cancer - 34.0% baseline vs. 46.5% follow-up - Colorectal cancer - 72.1% baseline vs. 84.5% follow-up - Breast cancer - 14.2% baseline vs. 20.0% follow-up | *Cervical* (p < 0.001),  *Colorectal* (p<0.001),  *Breast (*p=0.07) |
| Callison et al [17] | Moderate | Screening uptake | *Colorectal-cancer screening*  Adjusted model: likelihood of screening for colorectal cancer in the exposed metropolitan areas (MSAs) was 1.31 percentage points higher at 12 months. | 95% CI, 0.28 to 2.34 |
|  |  |  | *Colorectal-cancer screening*  Adjusted model: likelihood of screening for 24-month colorectal cancer screening in the exposed MSAs - 1.56 percentage points | 95% CI, 0.33 to 2.79 |
|  |  |  | *Breast Cancer Screening (mammography)*  Adjusted model: likelihood of screening for 12-month mammography in the exposed MSAs - 1.22 percentage points | 95% CI, 0.20 to 2.64 |
|  |  |  | Adjusted model: likelihood of screening for 24-month mammography in the exposed MSAs - 2.07 percentage points | 95% CI, 0.15 to 4.00 |
| Eljack et al [21] | Moderate | Knowledge | The educational program improved knowledge only; most women were willing to take the test. | – |
| Esmat Heydari, Azita Noroozi [23] | Moderate | Knowledge | *Multimedia education on mammography: (Pre- & Post-test)* Increase in knowledge | p < 0.001 |
|  |  |  | *Multimedia education on mammography: (Pre- & Post-test)* Perceived barriers | p = 0.007 |
|  |  |  | *Multimedia education on mammography: (Pre- & Post-test)* Perceived susceptibility, severity, benefit of mammography and health motivation | p > 0.05 |
|  |  |  | *Group education*: *(Pre- & Post-test) -* Knowledge, health motivation, perceived benefit, and barriers constructs, as the barriers decreased – statistically significant changes | p < 0.006 |
|  |  |  | *Group education*: *(Pre- & Post-test) –* Perception of benefits of mammography - higher | p = 0.003 |
| Hing et al * [33] | Moderate | Knowledge | *Domain 1: knowledge of breast cancer*  Respondents displayed a significant improvement in 16 out of 27 questions after intervention (i.e. health talk) | p < 0.05 |
|  |  |  | *Domain 2: knowledge of breast cancer screening*  significant improvement in most areas post intervention. | p < 0.05 |
|  |  |  | *Domain 3: attitudes & perception of breast cancer screening and treatment*  Significant improvement post intervention - from 65.6 to 84.7%. | p < 0.05 |
| Ma et al [14] | Moderate | Knowledge | *Changes in health beliefs and barriers*  Significant changes occurred in 8 of the 10 items. | – |
|  |  |  | *Post-intervention mammogram screening behavior*  Intention-to-treat analysis (assuming that follow-up non-completers did not take mammogram), the screening rates were 69% versus 4.1%. | p *< 0.001* |
| Ozerdogan et al [22] | Moderate | Knowledge | Women’s knowledge levels before the training on breast cancer (pre-test) and their knowledge after the training (post-test) – significant change | p = 0.001 |
| Rafie et al [24] | Moderate | Knowledge | Health belief category composite scores among those who completed both the pre-and post-health beliefs survey – significant increase in self-efficacy. | 4.01±0.79 vs 3.74±0.78,  p = 0.02 |
|  |  |  | No significant change in the other 4 categories. | – |

*Workplace related to health service

## Appendix J. Effectiveness of workplace cancer screening uptake interventions (Results as in original articles)

| **Study ID** | **Quality Score** | **Changes Screening Uptake** | **Results** | **Statistical Results** |
| --- | --- | --- | --- | --- |
| Bernstein et al * [15] | High | Screening Uptake | None of the incentive delivery mechanisms had a significant association with cancer screening participation.  Delivery mechanisms  CF: Client fulfilled (Clients determine specific rewards)  HAS: Health savings account  PR: Premium reduction  GC: Gift card | *Breast*  CF: 1.90 (0.57–6.35)  HAS: 1.07 (1.00–1.15)  PR: 1.78 (0.91–3.49)  GF: 1.89 (1.51–2.35)  *Cervical*  CF: 1.01 (0.49–2.09)  HAS: 1.04 (0.97–1.11)  GC: 0.93 (0.72–1.22)  *Colorectal*  HAS: 0.95 (0.89–1.01)  PR: 0.96 (0.50–1.84)  GC: 1.36 (1.05–1.75) |
| Cuellar et al [19] | High | Screening Uptake | Mammography - 2.7 percentage-point increase with financial incentives | p < 0.05 |
|  |  |  | Colorectal cancer screening - 2.2 percentage-point increase with financial incentives | p < 0.01 |
|  |  |  | Cervical cancer screening rates over time (Difference between Intervention and Control Groups) | No differences |
| Hui et al [8] | High | Screening Uptake | Improvement in previously non-adherent participants  Breast cancer screening – 52.4%  Cervical cancer screening – 41.3%  Colorectal cancer screening – 33.5% | – |
| Shepherd et al [26] | High | Screening Uptake | Population-based outreach  Screening rate – 167 out of 840 participants (19.9%) received the tests; Out of 167 participants, 89 (53.3%) completed | – |
|  |  |  | Office visit-based outreach  Screening rate – 56 out of 68 participants received the tests; Out of 56 participants, 43 (76.8%) completed | – |
|  |  |  | Office visit -based outreach had higher screening rate than population-based outreach (76.8% vs 53.3%). | p < 0.001 |
| Warner et al [32] | High | Screening Uptake | *Cervical cancer screening*  Papanicolaou – Pre-intervention - 77.7%, Post intervention - 79.9% | Δ_pre-post_: 2.2%; (p= 0.28) |
|  |  |  | *Breast cancer screening*  Mammogram – Pre-intervention - 66.4%, Post intervention - 68.5% | Δ_pre-post_: 2.2%; (p= 0.47) |
|  |  |  | *Colorectal cancer screening*  Sigmoidoscopy – Pre-intervention - 0.9%, Post intervention - 1.8%  Colonoscopy – Pre-intervention - 30.8%, Post intervention - 30.8%  FIT testing – 13.8% at baseline to 56.9% (p<0.001) | Sigmoidoscopy Δ_pre-post_: 0.9%; (p= 0.56),  Colonoscopy Δ_pre-post_: 0%; (p= 1.00),  FIT Δ_pre-post_: 43.1%; (p < 0.001) |
| Bardach et al [11] | Moderate | Screening Uptake | *Colorectal cancer screening* (Immunochemical fecal occult blood test/iFOBT)  Control period – 8.4 per 10,000 employees  Intervention period – 130.5 per 10,000 employees | p < 0.001 |
| Hannon et al * [31] | Moderate | Screening Uptake | *Colorectal cancer screening*  Employees receiving test kit during implementation - 8.7%  Employees screening rate - 4.4% upon receiving test kit | – |
| Jensen et al * [10] | Moderate | Screening Uptake | *Colorectal cancer screening*  Stock vs narrative condition – 8.9% increase in narrative condition | p < .05 |
|  |  |  | Non-narrative conditions vs narrative conditions - 9.2% increase in colonoscopy screening in narrative conditions. | p <.05 |
|  |  |  | Tailoring did not increase screening behavior overall. | – |
| Ma et al [14] | Moderate | Screening Uptake | *Breast cancer screening* (Mammography)  Baseline - 10.3%  Intervention group - 72.6% at 6-month follow-up | Δ_baseline-follow-up_: 62.3%  (p < 0.001) |
| O'Keefe et al [20] | Moderate | Screening Uptake | University of Alabama in Huntsville  FITs distribution - 86 test kits, return rate – 62 out of 86 (72.1%)  University of South Alabama  FITs distribution - 146 test kits, return rate – 111 out of 146 (72.1%) | – |
| Ozerdogan et al [22] | Moderate | Screening Uptake | Invited for participation – 903 women  Participants in training program – 405 out of 903  Eligibility among participants – 144 out of 405  Current with screening recommendation – 55 out of 144  Screening rate – 45 out of 144 | – |
| Rafie et al [24] | Moderate | Screening Uptake | *Colorectal cancer screening* rate – 20.6% increase (52.7 to 73.3%) | 20.6% increase in screening rate |
| Shima et al [28] | Moderate | Screening Uptake | *Breast cancer screening*  Intervention group – 259 (53.1%) of participants  Control group – 67 (7.4%) of participants | Adjusted OR: 14.22 (95% CI: 8.97– 22.54) |
| Ueda et al [30] | Moderate | Screening Uptake | *Cervical cancer screening*  Eligible participants – 681  Registration of HPV test - 205 (30%) out of 681  Screening rate – 128 out of 681 (19%) | – |

*Workplace related to health service

| Appendix K. Studies excluded from the analysis (Results as in original studies) | | | | |
| --- | --- | --- | --- | --- |
| **Study ID** | **Quality of studies** | **Risk of bias of studies** | **Intervention** | **Results as in original studies** |
| Greenwald et al [27] | Moderate | Serious risk of Bias | Educational program for colorectal cancer | Having insurance was positively associated with discussing colorectal cancer screening with doctors and family. |
| Hafeez et al [29] | Low | Serious risk of Bias | Educational program for cervical cancer to registered nurses | Knowledge and practice of cancer screening increased while their attitude on taking screening tests did not change. |
| Ahmed et al [9] | Low | Moderate risk of bias | Educational program for cervical cancer | There mean total knowledge score rose from 19.1±14.7 at  baseline to 35.3±9.3 post-intervention (p<0.0001). There  was highly significant increase in scores of HBM  components post-intervention (p<0.0001), with the total  score increasing from 26.43±6.18 to 37.91±5.52. There were 15(4.2%) subjects with high level of knowledge,  33(9.2%) moderate and 312(86.7%) low at baseline. The  corresponding data post-intervention was 22(6.1%),  148(41.1%) and 190(52.8%). Likewise, 5(1.4%) subjects had  high level of beliefs, 213(59.2%) had moderate and  142(39.4%) had low at baseline. The corresponding data  post-intervention was 125(34.7%), 215(59.7%) and  20(5.6%). |
| Kim et al [12] | Low | Moderate risk of bias | Providing FIT test kits for colorectal cancer | 25.6% increase in screening rate from the previous year was seen. |
| McFall et al [18] | Low | Serious risk of Bias | Sending reminders for colorectal cancer screening | 16% increase in screening rate from baseline was seen. |
| Matsuura et al [16] | Low | Serious risk of Bias | Providing cervical cancer screening | 36% of the target population took up the service. |

**References:**

1. Siu AL: **Screening for Breast Cancer: U.S. Preventive Services Task Force Recommendation Statement**. *Ann Intern Med* 2016, **164**(4):279-296.

2. US Preventive Services Task Force: **Screening for Breast Cancer: US Preventive Services Task Force Recommendation Statement**. *JAMA* 2024.

3. Grossman DC, Curry SJ, Owens DK, Bibbins-Domingo K, Caughey AB, Davidson KW, Doubeni CA, Ebell M, Epling JW, Jr., Kemper AR *et al*: **Screening for Prostate Cancer: US Preventive Services Task Force Recommendation Statement**. *JAMA* 2018, **319**(18):1901-1913.

4. Krist AH, Davidson KW, Mangione CM, Barry MJ, Cabana M, Caughey AB, Davis EM, Donahue KE, Doubeni CA, Kubik M *et al*: **Screening for Lung Cancer: US Preventive Services Task Force Recommendation Statement**. *JAMA* 2021, **325**(10):962-970.

5. Davidson KW, Barry MJ, Mangione CM, Cabana M, Caughey AB, Davis EM, Donahue KE, Doubeni CA, Krist AH, Kubik M *et al*: **Screening for Colorectal Cancer: US Preventive Services Task Force Recommendation Statement**. *JAMA* 2021, **325**(19):1965-1977.

6. Curry SJ, Krist AH, Owens DK, Barry MJ, Caughey AB, Davidson KW, Doubeni CA, Epling JW, Jr., Kemper AR, Kubik M *et al*: **Screening for Cervical Cancer: US Preventive Services Task Force Recommendation Statement**. *JAMA* 2018, **320**(7):674-686.

7. Abdullah F, O'Rorke M, Murray L, Su TT: **Evaluation of a worksite cervical screening initiative to increase Pap smear uptake in Malaysia: a cluster randomized controlled trial**. *Biomed Res Int* 2013, **2013**:572126.

8. Hui SK, Engelman KK, Shireman TI, Ellerbeck EF: **Adherence to cancer screening guidelines and predictors of improvement among participants in the Kansas State Employee Wellness Program**. *Prev Chronic Dis* 2013, **10**:E115.

9. Abdelmonsef Ahmed HA, Ibrahim Yassin SY, Mohamed Ahmed MS, Yousof Ali HZ: **Application Of Health Belief Model About Cervical Cancer Screening Among Female Officer Employees In Kafr-El Sheikh University, Egypt**. *J Pak Med Assoc* 2023, **73(Suppl 4)**(4):S67-S71.

10. Jensen JD, King AJ, Carcioppolo N, Krakow M, Samadder NJ, Morgan S: **Comparing tailored and narrative worksite interventions at increasing colonoscopy adherence in adults 50-75: a randomized controlled trial**. *Soc Sci Med* 2014, **104**:31-40.

11. Bardach AE, Pichon-Riviere A, Gibbons L, Alonso JP, Virgilio SA, Belizan M, Comolli M, Gonzalez LA: **Implementing Strategies at the Workplace Level to Increase Colorectal Cancer Screening Uptake in Argentina: A Controlled Interrupted Time-series Study**. *Cancer Prev Res (Phila)* 2022, **15**(5):335-345.

12. Kim JS, Bakr O, Xu M, Hochman M, Pagan VM: **Improving Colorectal Cancer Screening at an Employer-Sponsored Health Plan During the COVID-19 Pandemic**. *J Gen Intern Med* 2022, **37**(5):1334-1336.

13. Behnke AL, Krings A, Wormenor CM, Dunyo P, Kaufmann AM, Amuah JE: **Female health-care providers' advocacy of self-sampling after participating in a workplace program for cervical cancer screening in Ghana: a mixed-methods study**. *Glob Health Action* 2020, **13**(1):1838240.

14. Ma GX, Yin L, Gao W, Tan Y, Liu R, Fang C, Ma XS: **Workplace-based breast cancer screening intervention in china**. *Cancer Epidemiol Biomarkers Prev* 2012, **21**(2):358-367.

15. Bernstein A, Liang A, Luo F, Mann E, Shilts E, Serxner S: **Financial Incentives and Employer-Sponsored Health Activities**. *J Occup Environ Med* 2020, **62**(11):922-929.

16. Matsuura Y, Yoshioka M, Nakata A, Haraga M, Hachisuga T, Mori K: **Trends in Uterine Cervical Cancer Screening at Physical Health Checkups for Company Employees in Japan**. *J UOEH* 2019, **41**(3):327-333.

17. Callison K, Pesko MF, Phillips S, Sosa JA: **Cancer Screening after the Adoption of Paid-Sick-Leave Mandates**. *N Engl J Med* 2023, **388**(9):824-832.

18. McFall AM, Ryan JE, Hager P: **Implementing a client reminder intervention for colorectal cancer screening at a health insurance worksite**. *Prev Chronic Dis* 2014, **11**:E20.

19. Cuellar A, Haviland AM, Richards-Shubik S, LoSasso AT, Atwood A, Wolfendale H, Shah M, Volpp KG: **Boosting Workplace Wellness Programs With Financial Incentives**. *Am J Manag Care* 2017, **23**(10):604-610.

20. O'Keefe LC, Sullivan MM, McPhail A, Van Buren K, Dewberry N: **Screening for Colorectal Cancer at the Worksite**. *Workplace Health Saf* 2018, **66**(4):183-190.

21. Eljack A, Al Thani M, RE S: **Impact of health education on utilization of cervical cancer screening services among females working in secondary schools in Doha**. *Middle East Journal of Family Medicine* 2012, **10**(4).

22. Ozerdogan N, Sahin BM, Kosgeroglu N, Culha I, Celik N, Sayiner FD, Acikgoz A, Ozkaraman A, Arslantas D, Oner S *et al*: **Educational Study to Increase Breast Cancer Knowledge Level and Scanning Participation among Women Working at a University**. *Eur J Breast Health* 2017, **13**(3):113-116.

23. Heydari E, Noroozi A: **Comparison of Two Different Educational Methods for Teachers' Mammography Based on the Health Belief Model**. *Asian Pac J Cancer Prev* 2015, **16**(16):6981-6986.

24. Rafie CL, Hauser L, Michos J, Pinsky J: **Creating a Workplace Culture of Preventive Health: Process and Outcomes of the Colon Cancer-Free Zone at Virginia Cooperative Extension**. *J Cancer Educ* 2020, **35**(6):1135-1140.

25. Fernandez-Esquer ME, Nguyen FM, Atkinson JS, Le YC, Chen S, Huynh TN, Schick V: **Suc Khoe la Hanh Phuc (Health is Happiness): promoting mammography and pap test adherence among Vietnamese nail salon workers**. *Women Health* 2020, **60**(10):1206-1217.

26. Shepherd ME, Lecorps A, Harris-Shapiro J, Miller-Wilson LA: **Evaluating Outreach Methods for Multi-Target Stool DNA Test for Colorectal Cancer Screening Among an Employer Population**. *J Prim Care Community Health* 2021, **12**:21501327211037892.

27. Greenwald BJ, Edwards JU: **Worksite education programs by county extension agents to promote colorectal cancer prevention and screening**. *Gastroenterol Nurs* 2010, **33**(5):348-352.

28. Shima A, Tanaka H, Okamura T, Nishikawa T, Morino A, Godai K, Tatsumi Y, Kawahara M, Kiyohara M, Kawatsu Y *et al*: **Offering on-site mammography in workplaces improved screening rates: Cluster randomized controlled trial**. *J Occup Health* 2023, **65**(1):e12389.

29. Hafeez R, Perveen F, Zafar SN, Hafeez A: **Educational effect on knowledge, attitude and practice among registered nurses regarding cervical cancer, its prevention and screening in Krachi, Parkistan**. *Journal of Pakistan Medical Association* 2020, **70**(4).

30. Ueda Y, Kawana K, Yanaihara N, Banno K, Chhit M, Uy K, Kruy L, Sann CS, Ishioka-Kanda M, Akaba H *et al*: **Development and evaluation of a cervical cancer screening system in Cambodia: A collaborative project of the Cambodian Society of Gynecology and Obstetrics and Japan Society of Obstetrics and Gynecology**. *J Obstet Gynaecol Res* 2019, **45**(7):1260-1267.

31. Hannon PA, Vu T, Ogdon S, Fleury EM, Yette E, Wittenberg R, Celedonia M, Bowen DJ: **Implementation and process evaluation of a workplace colorectal cancer screening program in eastern Washington**. *Health Promot Pract* 2013, **14**(2):220-227.

32. Warner EL, Martel L, Ou JY, Nam GE, Carbajal-Salisbury S, Fuentes V, Kirchhoff AC, Kepka D: **A Workplace-Based Intervention to Improve Awareness, Knowledge, and Utilization of Breast, Cervical, and Colorectal Cancer Screenings Among Latino Service and Manual Labor Employees in Utah**. *J Community Health* 2019, **44**(2):256-264.

33. Hing JJX, Lee WP, Chua YNS, Tan PT, Mok CW, Sudhakar SS, Seah CM, Tan SM: **Impact of health talks on knowledge, attitudes and perception of breast cancer screening and treatment amongst healthcare staff by a breast surgical unit in a public healthcare institution: a cross-sectional study**. *BMC Womens Health* 2021, **21**(1):308.
